# Sacral neuromodulation treatment for urinary voiding dysfunctions: results of treatment with the largest single-center series in a tertiary referral center in Turkey

**DOI:** 10.55730/1300-0144.5575

**Published:** 2022-03-19

**Authors:** Umut KÜTÜKOĞLU, Türker ALTUNTAŞ, Bahadır ŞAHİN, Rahmi ONUR

**Affiliations:** Department of Urology, Faculty of Medicine, Marmara University, İstanbul, Turkey

**Keywords:** Sacral neuromodulation, efficacy, voiding dysfunction, treatment

## Abstract

**Background/aim:**

Sacral neuromodulation (SNM) is a minimally invasive treatment that modulates spinal reflexes to regulate bladder, urinary sphincter, and pelvic floor and has successfully been used in the treatment of refractory voiding dysfunctions. The aim of this study was to present our experience with SNM in a tertiary referral center with the largest number of patients and review the safety and efficacy of the procedure.

**Materials and methods:**

A total of 42 patients with refractory lower urinary tract symptoms were included into the study. After an initial test period, patients who showed more than 50% improvement in their symptoms underwent the second stage of SNM. Twelve patients had overactive bladder (OAB), bladder pain syndrome/interstitial cystitis (BPS/IC) and 17 had urinary retention. The clinical success was examined during follow-up by voiding diary, urodynamics, and global response assessment.

**Results:**

Between February 2015 and December 2020, a total of 29 patients underwent stages I&II SNM procedures. The mean ages of patients in OAB/BPS group and retention group were 40 (37–57 years) and 35 (27–44 years), respectively. Mean follow-up time was at least 1 year. Overall, 58.5% success rate was observed in OAB, BPS/IC, and urinary retention groups. Global response assessment score in both groups increased significantly (p = 0.001). No statistically significant difference was found between success or failure rates when sex and age were variable parameters (p > 0.05).

**Conclusion:**

SNM appears to be an effective and safe treatment option in restoring voiding dysfunctions in patients with refractory idiopathic and neurogenic voiding dysfunctions. Our initial series revealed favorable results; however, further studies with larger series and longer follow-up are needed.

## 1. Introduction

Sacral neuromodulation (SNM) has become an established treatment modality for refractory lower urinary tract symptoms and has been approved by FDA for the treatment of frequency-urgency syndrome, idiopathic urinary retention, and for some bowel dysfunctions. In urology, it is widely used as a third- or fourth-line treatment in patients with overactive bladder, bladder pain syndrome/interstitial cystitis, and neurogenic lower urinary tract dysfunction [1,2]. Although the exact mechanism of action of SNM is not fully understood, it is postulated that chronic stimulation of sacral spinal nerve root modulates the spinal reflexes that influence the bladder and bowel and reverses the aberrant neural activity [3,4]. The procedure is performed in two stages. Initially, peripheral nerve evaluation by a monopolar, temporary lead, or a quadripolar (tined) lead is used for test period [4]. This test period or stage 1 procedure evaluates whether the patient has a significant improvement which is denoted as 50% or more improvement in her or his symptoms. A battery-powered or rechargeable implantable pulse generator is implanted in patients who benefited the first stage [2,5–6].

The safety and efficacy of SNM has been well-documented in the literature. Success rate of use of SNM for refractory OAB has been reported to differ between 64% and 88% [7]. A systematic review revealed 39% cure rate and 67% improvement of 50% or greater in symptoms of patients [1]. Because of ineffectiveness of medical agents and hesitancy for self-catheterization in idiopathic chronic urinary retention patients, SNM became a very attractive alternative in this subgroup of patients. A metaanalysis revealed that both unilateral and bilateral SNM were effective for the treatment of urinary retention [1]. Despite the established efficacy of SNM for treatment of OAB and urinary retention, there is insufficient evidence for its role in the management of chronic pelvic pain, interstitial cystitis, and sexual dysfunction [8]. Moreover, SNM is not an easily accessible treatment modality in every country because of high-cost, staged implantation technique, and reimbursement issues. Those limitations are especially important in developing world countries and in patient care systems where government health care systems are in charge. SNM has been approved and has been reimbursed by health care system in Turkey since 2007. Five to six years of paucity occurred but since 2013, it has been reimbursed again. In this study, we aimed to report our experience with SNM as the largest single-center results and review the safety and efficacy of the procedure in patients with refractory lower urinary tract dysfunctions.

## 2. Materials and methods

**Figure 1:**
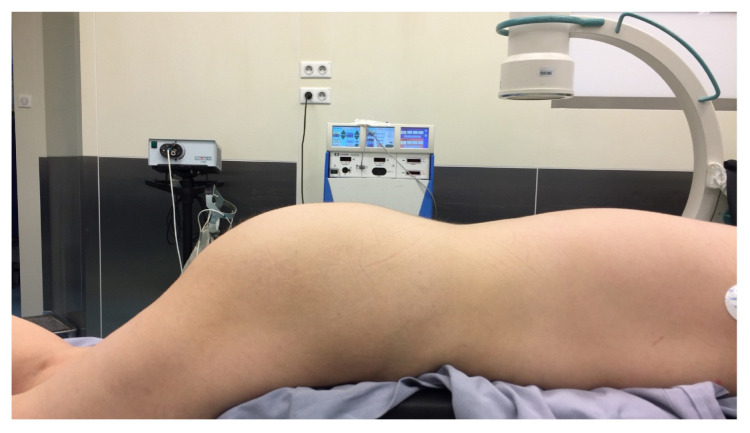
Operation position.

**Figure 2:**
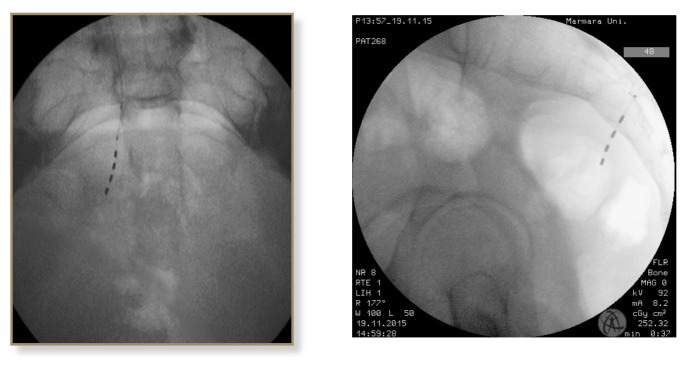
(a,b) Introduction of lead under fluoroscopic guidance.

**Figure 3:**
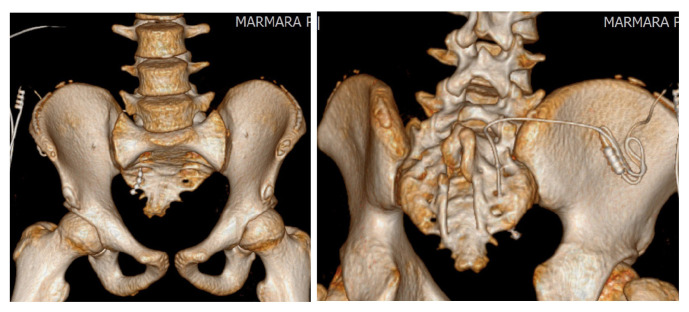
(a,b) Confirmation of lead location under 3D computerized tomography.

Between February 2015 and December 2020, a total of 42 patients underwent stage I tined lead electrode placement and stage II intermittent pulse generator (IPG) (Medtronic Inc., Fridley, MH, USA) therapy were performed in patients who showed more than 50% improvement in their lower urinary tract symptoms after test period. Patients with refractory overactive bladder syndrome (OAB), urinary frequency-urgency, urge incontinence, idiopathic urinary retention, bladder pain syndrome/interstitial cystitis (BPS/IC) and fecal incontinence resistant to conventional treatments were included into the study. Written informed consent was obtained from all patients. Exclusion criteria were pregnancy, urinary tract infection, bladder outlet obstruction, urinary tract malignancy, fistula, severe prolapse, active psychiatric disorder, and low bladder compliance. Institutional review board approval was obtained, and we retrospectively reviewed the charts of all patients and recorded the outcome of SNM procedure. Patients with symptom improvement of at least 50% or more within the test period underwent stage II procedure with placement of intermittent pulse generator (IPG). Sacral electrode was placed (stage I) to sacral spinal nerve 3 (S3) and IPG implantation (stage II) was performed in successful cases by placing the battery into the buttock area as described previously [9]. After placing the patient to prone position, lumbar lordosis was reduced as much as possible by supporting the pelvic ring and tilting the operating table (Figure 1). Identifying the medial aspect of S3 foramen standard-length (9 cm, 20 gauge) were introduced by an angle of 60 degrees 1.5–2 cm above the marked skin point. These steps were performed under fluoroscopy (Figures 2a and 2b). Once the foramen entered, stimulation is performed and motor responses of bellow and plantar flexion were observed. Ideally, testing at a low amplitude, i.e. <2mA and getting motor responses will probably reveal the same efficacy at lower thresholds (i.e. 1 mA) when patient is awake. Final position of tined lead electrode is achieved by using an introducer under lateral view with continuous fluoroscopy (Figure 2b). In difficult cases, patients with complex bony structures and history of bone surgeries, we further obtained 3-dimensional (3D) computed tomography images to see and confirm the exact location of the electrode (Figures 3a and 3b).

Demographic data of all patients were recorded. A detailed history and urogenital and neurological examinations were performed. Voiding diary, routine laboratory examination, and urodynamic investigation were conducted. Etiology of the voiding dysfunction, symptom duration and follow-up period were noted. Patients were followed every 3 months after the procedure for the 1st year and then yearly. Percentage improvement in symptoms, median test period, voiding diaries, and global response assessment (GRA) scores were determined. Improvement of symptoms of at least 50% or more, discontinuation or significantly decreased use of clean intermittent catheterization, improvement of frequency, urgency and urge incontinence symptoms were considered success of the procedure. Patients were controlled for procedure-related complications and any other side effects.

### 2.1. Statistical analyses

Statistical analyses were performed using SPSS^®^ 22 software. Descriptive analyses were given as medians, and interquartile ranges were used. For dependent variables, Wilcoxon tests were used. Categorical variables were compared with chi-squared test between groups if the groups were independent. If the assumptions of chi-squared did not hold due to low expected cell counts, Fisher’s exact test was used for the comparison of categorical variables. For all statistical tests, a p-value below 0.05 was considered statistically significant and confidence intervals are set as 95%.

## 3. Results

Of the 42 patients, thirteen had no benefit during a mean test period of 26 days (5–33 days) and tined-lead electrode was removed under local anesthesia. Second stage was performed in 29 patients who showed more than 50% symptomatic improvement. Etiology included 12 (41%) patients who had diagnosis of OAB, BPS/IC, whereas 17 (59%) patients underwent SNM due to a diagnosis of detrusor insufficiency or urinary retention (Table 1).

Demographic data of the patients are listed in Table 1. Significantly higher numbers of female patients were observed in OAB, BPS/IC group, whereas male and female ratios were similar in patients with urinary retention. The median follow-up period was 36 months in the OAB/IC group, and 14 months in the urinary retention group.

Overall, we observed 58.5% success rate in our cohort with similar ratios in both OAB, BPS/IC patients and urinary retention groups (Table 2). There was only one patient in OAB, BPS/IC group with neurogenic lower urinary tract dysfunction, whereas 35% of the patients in urinary retention group had neurogenic etiology (Table 1). Although maximum cystometric capacity (MCC) increased in the first group and a 25% decrease in MCC was present in urinary retention group, no statistical significance was achieved (p > 0.05) (Table 2). Nevertheless, global response assessment score (GRA) in both groups increased significantly (p = 0.001) ( Table 2).

No statistically significant difference was found between success or failure rates when sex and age were variable parameters (p > 0.05). We also compared success rates of SNM with respect to etiology. Of the 10 patients who had idiopathic urinary retention, eight (80%) had successful outcome, whereas 2 (33%) patients with neurogenic etiology showed success. Despite the higher success rate in patients with idiopathic etiology, no statistically significant difference was reached (p = 0.145).

We initially revised the place of electrode in 5 patients who had loss of therapeutic efficacy. However, neither new lead placement nor placing the electrode to contralateral S3 foramen yielded successful results. Thus, we discontinued to revise electrode due to no additional benefit. Postoperative complications were uncommon. Three (10%) patients had pain on implant site and IPG was moved to another part in these patients. There was implant infection in one (3%) patient in whom all system was removed and one (3%) patient experienced lead migration and required revision.

## 4. Discussion

Sacral neuromodulation was approved in 1997 for treatment of urge incontinence and in 1999 for urgency/frequency syndrome and nonobstructive urinary retention [10]. Since then, it has successfully been used in treatment of many cases of refractory lower urinary tract dysfunctions. SNM has also off-label use in several other pathologies such as neurogenic lower urinary tract dysfunctions, interstitial cystitis/bladder pain syndrome, male and female sexual dysfunction, and chronic pelvic pain [11]. In this study, we presented our initial experience and follow-up data for SNM treatment with the highest number of patients in a single tertiary referral center in Turkey.

The European Association of Urology (EAU), Society of Urodynamics, Female Pelvic Medicine & Urogenital Reconstruction (SUFU) and several other organizations suggest SNM as third-line treatment option in the treatment of refractory voiding dysfunctions with the same priority as botulinum toxin [12, 13]. However, SNM has been considered a 4th-line treatment modality in Turkey and there are many restrictions to its use such as previous botulinum toxin failure, interstitial cystitis history of at least 5 years, no reimbursement for patients older than 55 years and patients with neurogenic etiology. Thus, during a period of 5 years, we could recruit only 42 patients who fulfilled all criteria in our study. Our data showed an overall 69% IPG implantation rate after a successful test period. Of the 16 patients with OAB/IC diagnosis, twelve patients (75%) underwent second stage, whereas seventeen (66%) out of 26 patients with urinary retention received IPG implantation. Hoag et al. reported the success rate for first-stage SNM in botulinum toxin naive patients as 70.2%. In patients who had failed botulinum toxin therapy, their first stage success rate was reported to be 63.9% [7]. Because of reimbursement restrictions, we also could perform SNM only on patients who previously failed botulinum toxin therapy. We had a mean duration of test phase of 26 days. Although test phase was kept shorter in most of the studies, Kessler et al. evaluated the role of prolonged test phase (18–29 days, median 28 days) and suggested prolonged sacral neuromodulation testing for accurate patient selection [14]. We also chose to prolong the test period to properly select the patients and obtain accurate responses in the meantime since many patients suffering from these chronic pathologies do usually have placebo effect at the beginning and reveal false positive responses. Despite the prolonged testing period, we had no increased rate of infection in our cohort.

Success rate of SNM in patients with OAB/IC was reported to be 67% in a systematic review [15]. Similarly, Peters et al. reported 70% success rate in both wet and dry OAB patients [16]. Noblett et al. have recently reported 77% success rate at 12 months postoperatively [17]. In our series, the success rate was 58% in OAB/IC group (Table 2). All patients in this group had intravesical botulinum toxin injection treatment previously and failed. Thus, previous botulinum toxin treatment failure in these patients might reflect a more resistant group of patients. In a similar study, Hoag et al. reported 64% success rate in patients for whom botulinum toxin treatment was proven to be unsatisfactory and considered failure [7].

Since pharmacotherapy has no or minimal benefit in treatment of urinary retention, SNM is the only viable alternative to catheterization in patients with detrusor insufficiency and/or chronic urinary retention. In a long-term prospective multicentric study, 71% of the patients with urinary retention showed successful outcome [18]. Similarly, in their early experience, out of 9 patients with poor emptying and/or retention, Al-Azzawi reported 67% therapeutic benefit from SNM treatment [19]. In another randomized controlled trial, 68 patients underwent SNM for urinary retention. At 18 months of follow-up, 75% of the patients did not require catheterization or had significant reduction in voided volume per catheterization [20]. In a metaanalysis conducted with 14 studies, data of 478 patients revealed that change in residual and voided urine volumes were significantly improved after SNM treatment favoring SNM (p < 0.00001). With a minimum follow-up of 6 months, it was concluded that SNM is effective for the treatment of chronic urinary retention [21]. However, in a recent case series, the cure rate in patients with non-Fowler’s idiopathic urinary retention, the cure rate was 54% [22]. In our series, we had 59% success rate for SNM in the treatment of poor emptying and/or urinary retention which was a relatively lower success rate. Possible explanations for this finding may be as follows: nearly one third of patients with urinary retention had congenital neurological problems and a long history of neural plasticity. Recently, it was hypothesized that SNM, initiated during the acute phase following spinal cord injury (SCI), can decrease bladder spasticity preserving bladder compliance, bladder volume, and low bladder filling pressures. An early implantation of intervention might have been more successful because once changes in the neurological control of the bladder have occurred following SCI, they are irreversible in most cases [23]. Thus, a low success rate (33%) of SNM in patients with long history of neurological etiology can be explained by irreversible changes that have occurred due to neurogenic bladder.

Similarly, it should be acknowledged that SNM has a limited role in the treatment algorithm of neurogenic LUT dysfunctions as stated by 2013 International Consultation on Incontinence [24]. Although EAU guidelines suggest a beneficial role of SNM for treating neurological symptoms, lack of randomized controlled trials and a lack of clarity as to which neurological cases would show benefit necessitates further research and limits its widespread use for treatment of neurogenic LUTD [25]. Thus, there are still concerns of SNM in patients with flaccid neurogenic bladder, especially due to lack of efficacy over time. It was reported that one-third of patients’ symptoms return to baseline levels in less than 4 years [26]. With use of tined-lead electrode, staged implantation and percutaneous technique, SNM has become a minimally invasive procedure with no major complications. The most common complications were reported to be infection, lead migration, pain on the IPG site, and attenuation of response [27]. Although Van Kerrebroeck et al. reported a high rate of SNM-related adverse events (67%); of the 102 patients, the device was explanted from only 16 patients due to adverse event or lack of efficacy [18]. In their short prospective series, Al-Azzawi et al. had 12.5% infection rate [19]. Rios et al. reported no significant complications. However, they had 10.5% rate of infection rate in their series and 2% pain at IPG site [28]. In our series we also had no significant complications. There was pain on IPG site in 5 patients (10%), 1% infection, and 1% lead migration in our cohort. Overall, 5 (17%) patients required lead revision due to loss or attenuation of efficacy. Since we performed all SNM procedures with general anesthesia in OR, this factor might have been contributed to correct marking of IPG site initially, forming proper tunnel with keeping strict sterilization of the surgical area.

The main limitations of our study were its retrospective nature and nonhomogenous patient profile. Similarly, we had a small number of patients in each group, though this cohort represents the largest series published to date in Turkey. Despite responses detected in treatment groups, we observed no statistically significant difference with respect to final outcomes. However, global response assessment revealed significant benefit of the SNM. Another limitation was presence of patients with a long history of neurogenic bladder and presence of irreversible changes in their bladders. A further placebo effect could also interfere with procedure outcome in those patients. Nevertheless, SNM is a viable and minimally invasive treatment option in symptomatic treatment of patients with refractory voiding dysfunctions with successful outcomes in selected patients. Our initial experience with the largest number of patients in Turkey revealed comparable results to the published literature.

## Figures and Tables

**Table 1:** Patient demographics and etiology of voiding dysfunctions in each group.

OAB/IC group		Urinary retention group	
Variable	Value	Variable	Value
**Total number of patients**	12	**Total number of patients**	17
**Sex, n (%)**		**Sex, n (%)**	
Male	2 (16%)	Male	8 (47%)
Female	10 (84%)	Female	9 (53%)
**Age, years, median (IQR)**	40 (37–57)	**Age, years, median (IQR)**	35 (27–44)
**Etiology n (%):**		**Etiology, n (%):**	
Overactive bladder	6 (50%)	Idiopathic	11(65%)
Interstitial cystitis	5 (41%)	Neurogenic	6 (35%)
Neurological disease	1 (9%)		
**Symptom duration, years, median (IQR)**	6 (4–13)	**Symptom duration, years, median (IQR)**	4 (2–6.5)
**Follow-up, month, median (IQR)**	36 (25–54)	**Follow–up, month, median (IQR)**	14 (7–24)

IQR: Interquartile range

Twelve (41%) patients had diagnosis of OAB, BPS/IC, whereas 17 (59%) patients underwent SNM due to a diagnosis of detrusor insufficiency or urinary retention. There was only one patient in OAB, BPS/IC group with neurogenic lower urinary tract dysfunction whereas 35% of the patients in urinary retention group had neurogenic etiology.

**Table 2:** Outcome analyses after sacral neuromodulation.

OAB/IC Group			Urinary Retention Group		
Variable	Value	p	Variable	Value	p
**Max cystometric capacity, mL, median (IQR)**			**Max cystometric Capacity, mL, median (IQR)**		
Before SNM	190 (155–360)		Before SNM	450 (345–625)	
After SNM	290 (180–320)	0.359	After SNM	490 (345–535)	0.224
**GRA, mean (SD)**			**GRA, mean (SD)**		
Before SNM	1 (1)		Before SNM	1 (1)	
After SNM	1–5 (3.45)	**0.007**	After SNM	1–5 (3.54)	**0.001**
**Final outcome, n (%)**			**Ending status, n (%)**		
Success	7 (58%)	0.417	Success	10 (59%)	0.407
Failure	5 (42%)		Failure	7(41%)	

OAB/BPS: Overactive bladder/Bladder pain syndrome, Max: Maximum, IQR: Interquartile range, SNM: Sacral neuromodulation, GRA: Global response assessment, SD: Standard deviation

We observed 58.5% success rate in our cohort with similar ratios in both OAB, BPS/IC patients and urinary retention groups. Maximum cystometric capacity (MCC) increased in the first group and a 25% decrease in MCC was present in urinary retention group, no statistical significance was achieved (p > 0.05) (Table 2). Nevertheless, global response assessment score (GRA) in both groups increased significantly (p = 0.001).
